# *Candida albicans SET3* Plays a Role in Early Biofilm Formation, Interaction With *Pseudomonas aeruginosa* and Virulence in *Caenorhabditis elegans*

**DOI:** 10.3389/fcimb.2021.680732

**Published:** 2021-06-10

**Authors:** Ruan Fourie, Jacobus Albertyn, Olihile Sebolai, Onele Gcilitshana, Carolina H. Pohl

**Affiliations:** Department of Microbiology and Biochemistry, University of the Free State, Bloemfontein, South Africa

**Keywords:** biofilm, *Candida albicans*, *Caenorhabditis elegans*, *Pseudomonas aeruginosa*, SET3

## Abstract

The yeast *Candida albicans* exhibits multiple morphologies dependent on environmental cues. *Candida albicans* biofilms are frequently polymicrobial, enabling interspecies interaction through proximity and contact. The interaction between *C. albicans* and the bacterium, *Pseudomonas aeruginosa*, is antagonistic *in vitro, with P. aeruginosa* repressing the yeast-to-hyphal switch in *C. albicans*. Previous transcriptional analysis of *C. albicans* in polymicrobial biofilms with *P. aeruginosa* revealed upregulation of genes involved in regulation of morphology and biofilm formation, including *SET3*, a component of the Set3/Hos2 histone deacetylase complex (Set3C). This prompted the question regarding the involvement of *SET3* in the interaction between *C. albicans* and *P. aeruginosa*, both *in vitro* and *in vivo.* We found that *SET3* may influence early biofilm formation by *C. albicans* and the interaction between *C. albicans* and *P. aeruginosa*. In addition, although deletion of *SET3* did not alter the morphology of *C. albicans* in the presence of *P. aeruginosa*, it did cause a reduction in virulence in a *Caenorhabditis elegans* infection model, even in the presence of *P. aeruginosa.*

## Introduction

Interkingdom interactions are ubiquitous in nature and can affect various aspects of the growth, antimicrobial resistance and virulence of species within a consortium (Peters et al., 2012; Stacy et al., 2016). In the opportunistic fungal pathogen, *Candida albicans*, these interactions are frequently encountered in polymicrobial associations formed with commensal microorganisms as well as pathobionts in humans (Morales and Hogan, 2010; Diaz et al., 2012; Neville et al., 2015). This is, in part, due to the ability to form biofilms on both abiotic and biotic surfaces (Polke et al., 2015).

*Candida albicans* exhibits polymorphism, with up to nine distinct phenotypes being formed (Noble et al., 2017). This includes the classical morphotypes - yeast, hyphae, pseudohyphae and chlamydospores - as well as non-classical phenotypes dependent on the expression of the white-opaque regulator, Wor1p (Lan et al., 2002; Pande et al., 2013; Tong et al., 2014; Noble et al., 2017). These different phenotypes show alterations in mode of growth, morphology, carbon source utilisation and virulence. Importantly, distinctive phenotypes also show differences in competitive fitness with resident or co-infecting bacteria and may alter the population dynamics of these bacteria (Pande et al., 2013; Fox et al., 2014).

*Candida albicans* is frequently co-isolated with the Gram-negative bacterium, *Pseudomonas aeruginosa* from the lungs of cystic fibrosis patients (Chotirmall et al., 2010; Leclair and Hogan, 2010; Haiko et al., 2019). *In vitro*, the interaction is characterised as antagonistic, with both species influencing each other (reviewed by Fourie and Pohl, 2019). The bacterium was found to lyse and kill hyphae of *C. albicans* through physical interaction (Hogan and Kolter, 2002; Brand et al., 2008; Bandara et al., 2010) and affects *C. albicans* biofilm formation and morphogenesis through various secreted factors and cell wall components (Kerr et al., 1999; Hogan et al., 2004; McAlester et al., 2008; Xu et al., 2008; Bandara et al., 2010; Holcombe et al., 2010; Reen et al., 2011; Bandara et al., 2013). This includes inhibition of morphogenesis from yeast to hyphal morphologies by phenazines, quorum sensing molecules, lipopolysaccharides and *via* sequestration of iron, and promotion of morphogenesis by peptidoglycan. These stimuli elicit their effects through various signalling pathways in *C. albicans*, including stimulation of morphogenesis through the mitogen activated protein (MAP) kinase signalling pathway and the cyclic adenosine monophosphate (cAMP)/protein kinase A (PKA), as well as repression by the transcriptional repressor, Tup1p (Shareck and Belhumeur, 2011). Therefore, multiple conflicting stimuli, occurring simultaneously, from co-incubation with *P. aeruginosa* may play a role to affect the morphology of *C. albicans*. Transcriptomic evaluation of *C. albicans* indicated the upregulation of *SET3* [a component of the Set3/Hos2 histone deacetylase complex (Set3C)] in the presence of *P. aeruginosa* (Fourie et al., 2021). This led to the question if Set3C may influence the interaction between *C. albicans* and *P. aeruginosa*, by integrating various external stimuli to influence biofilm morphology. As the contribution of this gene in the interaction of *C. albicans* with *P. aeruginosa* has not been evaluated before, its role in *in vitro* polymicrobial biofilm formation and virulence in *Caenorhabditis elegans* was examined.

## Materials and Methods

### Strain Maintenance

*Candida albicans* strains were stored at -80°C with 15% glycerol. Yeast strains were revived and maintained on yeast malt (YM) agar (3 g l^-1^ malt extract, 3 g l^-1^ yeast extract, 5 g l^-1^ peptone, 10 g l^-1^ glucose, 16 g l^-1^ agar) at 30°C. *Pseudomonas aeruginosa* PAO1 was stored at -80°C with 25% glycerol and revived/maintained on Luria-Bertani (LB) agar (5 g l^-1^ yeast extract, 10 g l^-1^ tryptone, 10 g l^-1^ sodium chloride and 15 g l^-1^ agar).

### Construction of Homozygous Deletion Mutants With CRISPR/Cas9

A published CRISPR-Cas9 system (Nguyen et al., 2017) was used for the construction of homozygous mutants for *SET3* with minor modifications. This method entails the introduction of a homozygous double stranded break at the site of interest and modification of the sites of interest with donor DNA in the wild type SC5314 strain. A detailed description of the procedure can be found in **Supplementary Text S1**. Following homozygous deletion of *SET3* yielding *set3*Δ/Δ, a complemented strain (*set3*Δ/Δ*::SET3)* was constructed by reintroduction of the wild-type gene by modified donor DNA.

### Preparation of Cells for Mono- and Polymicrobial Biofilms

#### Preparation of *C. albicans* Cells for Monomicrobial Biofilms

*Candida albicans* SC5314 (wild type) was grown on YM agar for 24 h at 30°C and was inoculated into 10 mL yeast nitrogen base (YNB) broth (10 g l^-1^ glucose, 16 g l^-1^ YNB) and incubated at 30°C for 24 h. Cells were harvested (1878 x g, 5 minutes) and the supernatant removed. This was followed by washing the cells twice with phosphate buffered saline (PBS) (Oxoid, England). The cells were counted with a hemocytometer and diluted to 1 x 10^6^ cells/mL in filter sterilized (0.22 μm nitrocellulose filter, Merck Millipore, Ireland) RPMI-1640 medium (Sigma-Aldrich, USA).

#### Preparation of *C. albicans* and *P. aeruginosa* Cells for Polymicrobial Biofilms

*Pseudomonas aeruginosa* PAO1 (wild type) was grown on LB plates for 24 h at 37°C. Cells were inoculated into 5 mL nutrient broth (1 g l^-1^ malt extract, 2 g l^-1^ yeast extract, 5 g l^-1^ peptone and 8 g l^-1^ sodium chloride) and incubated at 37°C for 24 h with shaking (150 rpm). These cells were washed (X3) and diluted to an optical density (OD_600_) of approximately 0.05 in RPMI-1640 medium containing 1 x 10^6^ cells/mL *C. albicans* (prepared as described in previous section).

### Quantification and Characterisation of Biofilm Formation

#### Biofilm Biomass of Mono- and Polymicrobial Biofilms

Cells were prepared as described above and 200 µL was dispensed into a 96-well plate (Corning Incorporated, Costar^®^, USA). The plate was incubated for 6h and 48h respectively at 37°C to allow the formation of biofilms. The crystal violet assay was performed on biofilms according to Jin and co-workers (2003) with minor modifications. Briefly, the supernatant from each well was removed and the biofilms were washed twice with sterile PBS. Biofilms were then left to air dry for 45 minutes and stained with 110 µL crystal violet (0.4% w/v; Merck, Germany) for 45 min (Jin et al., 2003). Biofilms were washed three times with 350 µL sterile H_2_O and de-stained with 200 µL 95% ethanol for 45 min. One hundred microliter of de-staining solution was then transferred to a clean 96-well plate and absorbance was measured at 595 nm. This experiment was performed in triplicate with four technical replicates per biological replicate.

#### Quantification of *C. albicans* and *P. aeruginosa* Colony Forming Units in Biofilms

Cells for mono- and polymicrobial biofilms were prepared as described above in flat-bottom 6 well culture plates (Corning Incorporated, USA) in 3 mL medium and incubated for 48h at 37°C to allow biofilm formation to take place. After incubation, biofilms were washed twice with sterile PBS, scraped off and suspended in PBS. Biofilms were then vortexed 3 times for 1 minute to remove adherent cells from one another (adapted from Fourie et al., 2017). For quantification of *C. albicans*, serially diluted cells were plated onto YM medium acidified with tartaric acid (final concentration 0.08%). For bacterial quantification, serially diluted cells were plated onto LB supplemented with 10 µg/mL amphotericin B (Sigma-Aldrich, USA) (Pires et al., 2013). Plates were incubated overnight, to allow formation of colonies, and counted. This experiment was performed in triplicate.

#### Morphology of Mono- and Polymicrobial Biofilms

Cells for mono- and polymicrobial biofilms were prepared as described above in flat-bottom 6 well culture plates in 3 mL medium (Fourie et al., 2017). After incubation for 24h at 37°C (to allow for mature biofilms without extensive killing by *P. aeruginosa*), supernatant was removed and approximately 5 mm rectangular sections of the wells were cut and placed in PBS. Cells were fixed overnight in 3% (v/v) glutardialdehyde (Merck, Germany) in phosphate buffer. This was followed by washing of biofilms with PBS and fixation with 1% osmium tetroxide (Merck, Germany) for 1h. Following fixation, biofilms were sequentially dehydrated with increasing concentration of ethanol (50% to 100%) and subjected to critical point drying (Samdri-795 Critical point dryer, Tousimis, USA). A gold layer was applied to the biofilms with a SEM coating system (Bio-Rad, UK) and examined using a JSM-7800F Extreme-resolution Analytical Field Emission SEM.

### Infection of *Caenorhabditis elegans* by *C. albicans* and *P. aeruginosa*

*Caenorhabditis elegans* AU37 [glp-4(bn2) I; sek-1(km4 X] (RRID : WB-STRAIN : WBStrain00000261), obtained for the *Caenorhabditis* Genetic Centre (University of Minnesota), was used for all infections. The nematodes were propagated and maintained on Nematode Growth Medium (3 g l^-1^ NaCl, 2.5 g l^-1^ peptone, 5 μg ml^-1^ cholesterol, 1 mM CaCl_2_, 1 mM MgSO_4_, 25 mM KPO_4_, 20 g l^-1^ agar) with *Escherichia coli* OP50 as a food source at 15°C (Brenner, 1974).

For infection by *C. albicans* alone, *C. albicans* strains were inoculated in YPD broth overnight at 30°C. Overnight cells were diluted to an OD_600_ of 0.8 and 100 μL was plated onto brain-heart infusion (BHI)-agar plates and incubated overnight at 30°C. Synchronised L4-stage nematodes were washed with M9 buffer (3 g l^-1^ KH_2_PO_4_, 6 g l^-1^ Na_2_PO_4_ and 1 mM MgSO_4_) and added to plates with *C. albicans*. Nematodes were incubated with *C. albicans* for 4 hours at 25°C and washed three times with M9 buffer to remove non-ingested *C. albicans* cells. Nematodes were then added at approximately 60 per well in a 6 well plate (Corning Incorporated, USA) containing 2 mL 80% M9 buffer and 20% BHI broth, with 90 μg/mL kanamycin and incubated at 25°C. Nematodes were monitored daily and dead nematodes (non-motile after mechanical stimulation with sterile pipette tip or penetration of *C. elegans* cuticle by *C. albicans* hyphae) were counted and removed.

For dual-infection by both *C. albicans* and *P. aeruginosa*, *C. albicans* was prepared on BHI-agar plates as described above. *P. aeruginosa* was inoculated in LB broth overnight and diluted to OD_600_ of 0.8. One hundred microliter of the *P. aeruginosa* suspension was plated on BHI-plates and incubated overnight at 37°C. Synchronised L4-stage nematodes were washed with M9 buffer and added to plates incubated with *C. albicans* for 2 hours at 25°C. Nematodes were washed three times with M9 buffer and added to BHI-plates with *P. aeruginosa* for 2 hours at 25°C. Nematodes were washed again with M9 buffer to remove non-ingested *P. aeruginosa* cells and placed in M9 buffer in 6 well plates (approximately 60 nematodes per well) and incubated at 25°C. Nematodes were monitored daily and dead worms (non-motile after mechanical stimulation with sterile pipette tip or penetration of *C. elegans* cuticle by *C. albicans* hyphae) were counted and removed. Nematodes incubated with *E. coli* OP50 prior to survival assay was used as a control. Infection was performed in triplicate for a total of 180 nematodes per strain of *C. albicans*. Survival metrics including Kaplan-Meier statistics, median survival time and log-rank test were performed with online application for survival analysis 2 (OASIS 2; Han et al., 2016).

### Statistical Analysis

Differences between control and experimental, as well as between *C. albicans* strains were determined with *t*-test, performed with GraphPad Prism version 7.00 for Windows (GraphPad Software, La Jolla California USA, www.graphpad.com) unless otherwise stated.

## Results

### Deletion of *SET3* Influences Early Biofilm Formation and Interaction With *P. aeruginosa*

**Figure 1:**
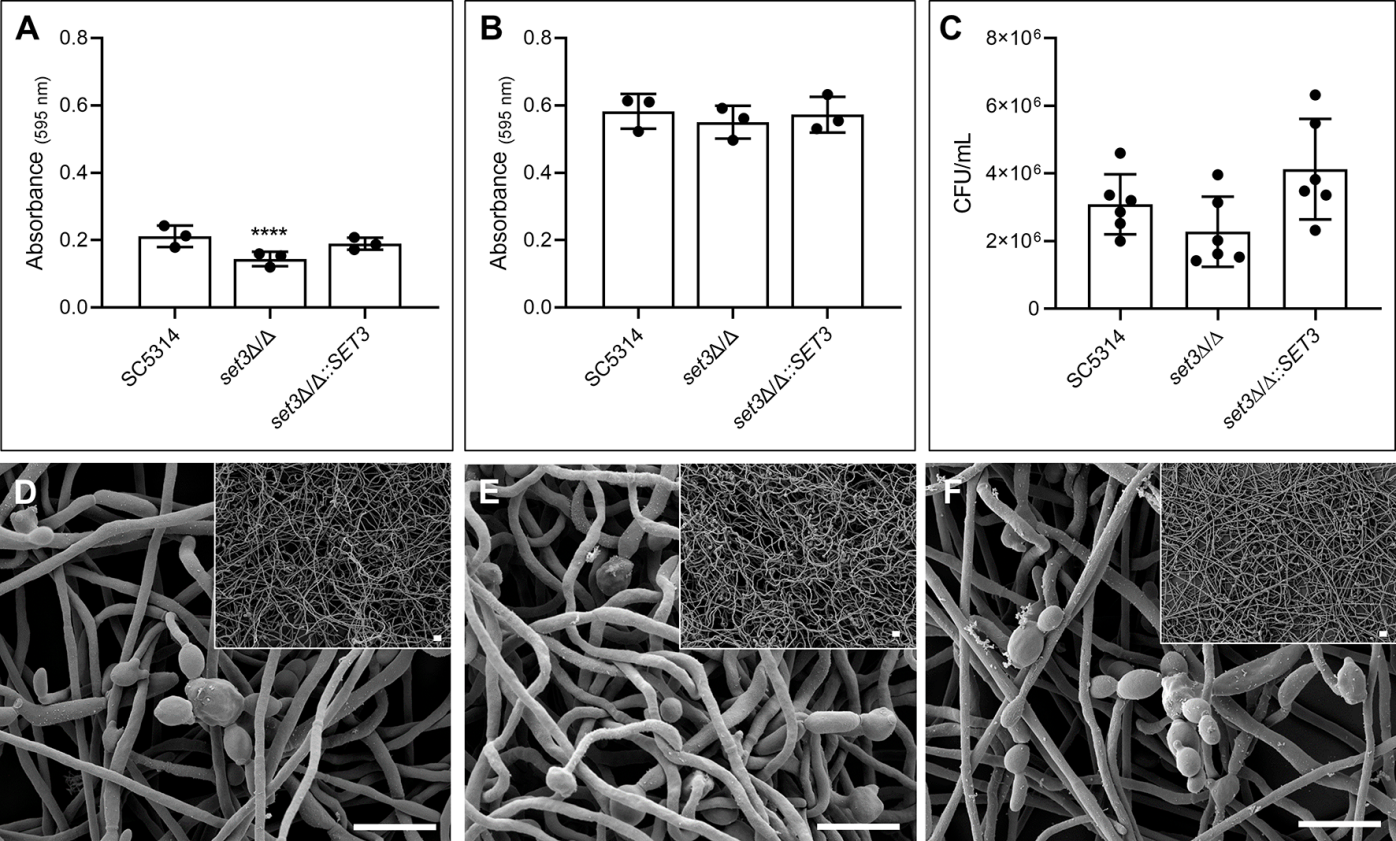
Effect of *SET3* deletion on *Candida albicans* monomicrobial biofilms. Monomicrobial biofilm biomass after 6 h **(A)** and 48 h **(B)** of the homozygous mutant of *SET3* (*set3*Δ/Δ) as well as the homozygous mutant with add-back of the wild-type gene (*set3*Δ/Δ*::SET3*). Colony forming units (CFU) of mature *C. albicans* biofilms are indicated in **(C)** as well as biofilm morphology of mature biofilms in **(D)** (SC5314), **(E)** (*set3*Δ/Δ) and **(F)** (*set3*Δ/Δ*::SET3*). Small panels on right corners indicate biofilms with lower magnification. Scale bars on all panels indicate 10 μm. *Significantly different from wild type (SC5314) (****P < 0.0001).

**Figure 2:**
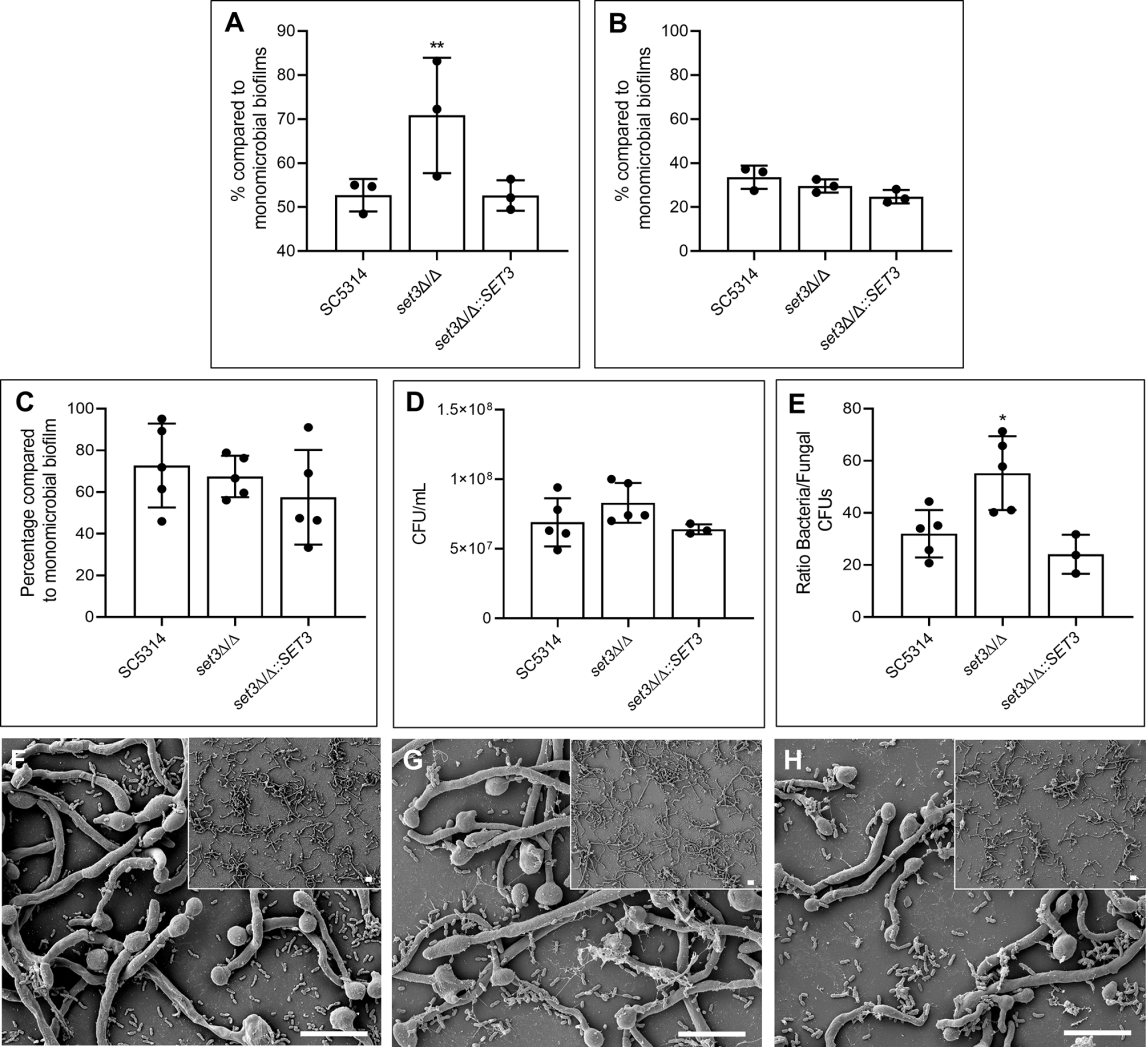
Effect of *SET3* deletion on *Candida albicans* polymicrobial biofilms with *Pseudomonas aeruginosa*. Polymicrobial biofilm biomass after 6 h **(A)** and 48 h **(B)** of the homozygous mutant of *SET3* (*set3*Δ/Δ) as well as the homozygous mutant with add-back of the wild-type gene (*set3*Δ/Δ*::SET3*) with *P. aeruginosa*. Colony forming units (CFU) of mature *C. albicans* biofilms are indicated in **(C)** and *P. aeruginosa* CFUs in **(D, E)** indicates the ratio of bacterial/fungal CFUs. **(F)** (SC5314), **(G)** (*set3*Δ/Δ) and **(H)** (*set3*Δ/Δ*::SET3*) indicates the morphology of mature polymicrobial biofilms. Small panels on right corners indicate biofilms with lower magnification. Scale bars on all panels indicate 10 μm. *Significantly different from wild type (SC5314) (*P < 0.05; **P < 0.005).

To evaluate the effect of the deletion of *SET3* on *C. albicans* biofilm formation, biomass production during early biofilm development (6h) as well as after 48 hours (mature biofilms) in both mono- and polymicrobial biofilms were quantified using the crystal violet assay. In addition, the population dynamics in polymicrobial biofilms were studied using CFUs of *C. albicans* and *P. aeruginosa*, as presented in **Figures 1**, **2**.

**Figure 1A** indicates a significant (*P* < 0.0001) reduction in biomass of early (6h) monomicrobial biofilms (approximately 33.6%) of *set3*Δ/Δ compared to the wild type. This was restored to wild type values with the re-introduction of the *SET3* gene. Mature (48h) biofilms of *set3*Δ/Δ did not exhibit this reduction in biomass (**Figure 1B**), and although a slight reduction in *set3*Δ/Δ CFUs is seen after 48 hours (**Figure 1C**), this is not statistically significant. In addition, **Figures 1D–F** indicate that the mature monomicrobial biofilms of *set3*Δ/Δ is composed of a thicker layer of hyphal cells (**Figure 1E**), confirming previous observations by Nobile et al. (2014).

When *set3*Δ/Δ was exposed to *P. aeruginosa* in a polymicrobial biofilm (**Figure 2**), a significant increase in polymicrobial biofilm biomass, compared to the polymicrobial biofilms with wild type *C. albicans*, is seen after 6h (**Figure 2A**). This effect could be restored to the wild type phenotype with the re-introduction of *SET3* and was lost during biofilm maturation (**Figure 2B**). Interestingly, although no statistically significant effect on *C. albicans* (**Figure 2C**) or *P. aeruginosa* (**Figure 2D**) CFUs is seen in the mature (48h) polymicrobial biofilms (due to the large variation between samples), a significant increase in the ratio of bacterial CFUs over *C. albicans* CFUs in polymicrobial biofilms of *set3*Δ/Δ is observed (**Figure 2E**). Deletion of *SET3* did not prevent the inhibition of *C. albicans* morphogenesis by *P. aeruginosa* (**Figures 2F–H**). However, longer hyphae are present in *set3*Δ/Δ polymicrobial biofilms (**Figure 2G** **)** than in either the wild type biofilms (**Figure 2F** **)** or the complemented strain (**Figure 2H**).

### *SET3* Influences Virulence of Mono- and Polymicrobial Infection in *C. elegans*

**Figure 3:**
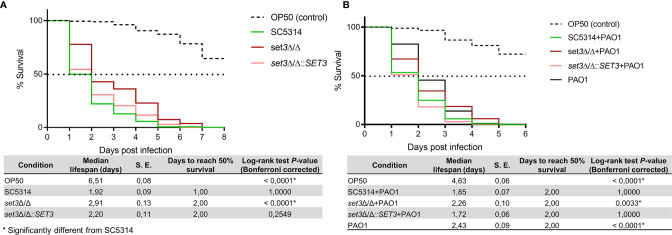
Survival of *Caenorhabditis elegans* infected with *Candida albicans* mutants and *Pseudomonas aeruginosa*. **(A)** - Percentage survival of *C. elegans* infected with *C. albicans* wild type (SC5314) or the homozygous mutant of *SET3* (*set3*Δ/Δ) as well as the homozygous mutant with add-back of the wild-type gene (*set3*Δ/Δ*::SET3*). **(B)** – Percentage survival of *C. elegans* co-infected with *C. albicans* mutants and *P. aeruginosa* (PAO1). Controls consists of *C. elegans* allowed to feed on *Escherichia coli* OP50 (OP50). The tables represent median lifespan with standard error (S. E.) along with days to reach 50% mortality. *P*-values are included for the Log-rank test for overall differences in survival. *Significantly different from wild type *C. albicans* (SC5314).

**Figure 3** indicates the percentage survival of *C. elegans* with survival statistics of *C. elegans* infected with *C. albicans* alone, or co-infected with *P. aeruginosa*. A significant (*P* < 0.0001) increase in survival of *C. elegans* infected with *set3*Δ/Δ compared to the wild type was found (**Figure 3A**). Virulence of *set3*Δ/Δ in *C. elegans* was restored through re-introduction of the wild-type gene (*set3*Δ/Δ*::SET3*). Notably, the deletion of *SET3* did not influence the ability of *C. albicans* to form hyphae and pierce the cuticle of *C. elegans*. Similar to single-species infection, co-infection by *C. albicans set3*Δ/Δ and *P. aeruginosa* also exhibited decreased virulence compared to the co-infection with the wild type (**Figure 3B**), indicating that Set3C contributes to virulence of *C. albicans* in *C. elegans*, even in the presence of *P. aeruginosa*.

## Discussion

In order to evaluate the possible role of *SET3* in the interaction between *C. albicans* and *P. aeruginosa*, the impact of homozygous deletion of *SET3* on biofilm formation of *C. albicans* was first evaluated. Using this approach, it was found that deletion of *SET3* negatively influence early biofilm formation (**Figure 1**), but that this effect was lost in mature biofilms, which also showed robust formation of hyphae after 48h, similar to results reported previously (Hnisz et al., 2010). Thus, although binding of the Set3C correlates with gene expression during morphogenesis, deletion of *SET3* leaves the expression of most genes unaffected (Hnisz et al., 2012) and may only transiently affect expression levels of key morphogenesis-related genes.

Upon evaluation of the effect of *SET3* deletion on the interaction between *C. albicans* and *P. aeruginosa*, an increase in biomass of the *set3*Δ/Δ polymicrobial biofilm was seen at 6h (**Figure 2**), however this effect was also lost upon maturation of the biofilms (48h). It must be noted that the previous upregulation of *SET3* in the presence of *P. aeruginosa* was also in 6h biofilms (Fourie et al., 2021), strengthening the finding that *SET3* may modulate early biofilm formation, especially in the presence of *P. aeruginosa*. Interestingly, although the Set3C may provide additional regulation, deletion of *SET3*, a core component of the complex, is unable to prevent the inhibition of *C. albicans* hyphal formation by *P. aeruginosa*. However, longer hyphae were observed, which may be as a result of the hypersensitivity of the cAMP/PKA signaling pathway previously reported for *set3*Δ/Δ (Hnisz et al., 2010). This indicates that *SET3* is required for the full wild type response of *C. albicans* to *P. aeruginosa*, mediating the morphological switch to the yeast morphology, which may allow dispersal and escape of *C. albicans* from the antagonistic effect of *P. aeruginosa.*

*Caenorhabditis elegans* is susceptible to *C. albicans* and *P. aeruginosa* (Tan et al., 1999; Pukkila-Worley et al., 2009) and is known to share a similar innate immune response with humans (Pukkila-Worley et al., 2011). The increased survival of nematodes infected with *set3*Δ/Δ (**Figure 3** **)** (even in the presence of *P. aeruginosa*) corroborates findings of reduced virulence of *set3*Δ/Δ as found previously in a murine model of systemic candidiasis (Hnisz et al., 2010) and further validates the use of this alternative infection model in the study of *C. albicans* virulence. *Candida albicans* primarily relies on hyphal formation to kill *C. elegans* in this assay (Pukkila-Worley et al., 2009). Notably, similar to the results in the murine model (Hnisz et al., 2010) in which hyperfilamentous *set3*Δ/Δ was less virulent, the deletion of *SET3* also did not decrease the ability of *C. albicans* to form hyphae and pierce the cuticle of *C. elegans.* This confirms that the reduction in virulence is not due to a lack of hyphae, even in this simpler model. This adds further complexity to the role of the regulation of morphogenesis in virulence. In mammals, the importance of a yeast phase during certain stages of dissemination *via* the blood stream, as well as the timing of the yeast to hyphal switch is considered crucial for virulence. However, further research needs to be done to better understand the role of the yeast phase in the *C. elegans* model, where infection does not spread *via* dissemination. Due to the low homology of the Set3C to human or other histone deacetylases, it deserves further attention as a therapeutic target as it may not only affect the virulence of *C. albicans* during single species infection, but also during polymicrobial infection with *P. aeruginosa.*

## Data Availability Statement

The original contributions presented in the study are included in the article/**Supplementary Material**. Further inquiries can be directed to the corresponding author.

## Author Contributions

RF and CP conceptualised study and RF performed experiments. JA provided scholarly input regarding mutant construction and RF and CP co-wrote the manuscript. OS and OG provided resources and edited the manuscript. All authors contributed to the article and approved the submitted version.

## Funding

This work was supported by the National Research foundation of South Africa [grant numbers 118543 and 115566 to CP and 121998 to OG].

## Conflict of Interest

The authors declare that the research was conducted in the absence of any commercial or financial relationships that could be construed as a potential conflict of interest.
