# Supplementary material for: Candida albicans SET3 Plays a Role in Early Biofilm Formation, Interaction With Pseudomonas aeruginosa and Virulence in Caenorhabditis elegans
Source: Front Cell Infect Microbiol. 2021 Jun 10;11:680732. doi: 10.3389/fcimb.2021.680732 (PMC8223063; doi:10.3389/fcimb.2021.680732)
Supplement: Supplementary file 1 [file DataSheet_1.docx]

### Construction of homozygous deletion mutants with CRISPR/Cas9

1. **Materials and methods**

The plasmids used in this study were obtained from Addgene (www.addgene.org). All primers were purchased from Integrated DNA Technologies and are listed in **Table S1**. All Polymerase chain reactions (PCRs) were performed with KAPA Hifi Hotstart PCR Kit (KAPA Biosystems Inc.) and colony PCR was performed with KAPA Taq PCR Kit (KAPA Biosystems Inc.), unless otherwise stated.

#### Plasmids and cassettes for deletion

Two microgram pADH99 (Addgene plasmid # 90979) was digested with PmeI (New England Biolabs, US) to release the *CAS9* cassette (**Figure S1A**). For the gRNA-cassette, the 5’ of the gRNA cassette was amplified from pADH110 (Addgene plasmid # 90982) with primers AHO1096-ver2 and AHO1098-ver2 (**Figure S1B**). To introduce target-specific CRISPR-sites into the gRNA cassette, gene sequences with approximately 500 bp upstream and downstream were obtained from *Candida* Genome Database (CGD) and CRISPR-sites (20 bp sequence followed by a protospacer adjacent motif, PAM) for *SET3* were retrieved with *Geneious* (version 10.2.6; https://www.geneious.com). CRISPR-sites were chosen as to have the highest on-site activity score (Doench *et al*., 2014) and off-target score (Hsu *et al*., 2013) compared to the diploid genome of *C. albicans* SC5314 (Muzzey *et al*., 2013; Skrzypek *et al*., “*Candida* Genome Database” http:candidagenome.org/ Accessed 20 July 2018) to minimize off target events in the *C. albicans* genome. In addition, CRISPR sites were chosen only if they were identical on both alleles of the diploid genome according to Assembly 22 of the *C. albicans* SC5314 genome (Muzzey *et al*., 2013; Skrzypek *et al*., “*Candida* Genome Database”). The flanking sequences, 5’-CGTAAACTATTTTTAATTTG-3’ and 5’-GTTTTAGAGCTAGAAATAGC-3’ were added to the 5’ and 3’ sides of the 20bp CRSIPR site without the PAM sequence respectively (**Table S4**). These two regions are complementary to *SNR52* promoter on pADH110 (Addgene plasmid # 90982) and structural gRNA sequence on pADH147 (Addgene plasmid # 90991) respectively. The 3’ of the gRNA cassette was constructed through amplification of pADH147 with the oligos with flanking sequences described above as forward primer (**Figure S1B**) and

**Table S1.** List of primers used in this study

| Name | Sequence (5’ to 3’) | Reference |
| --- | --- | --- |
| SET3-2F | GCAACATGAAATAGATGGCTG | This study |
| SET3-2R-overlap | CTGTCGAGGCAGTATCGACAAACCAAACAAACA | This study |
| SET3-3F-overlap | TGTCGATACTGCCTCGACAGTTACAGGAGAA | This study |
| SET3-3R | AGGCAAGAGACTGTGATTTGA | This study |
| SET3-CRISPR-1-deletion | CGTAAACTATTTTTAATTTGATACTTATGGAGCTTTTCCGGTTTTAGAGCTAGAAATAGC | This study |
| SET3-CRISPR-2-add-back | CGTAAACTATTTTTAATTTGGCTTCTTTACAACTTTAGCAGTTTTAGAGCTAGAAATAGC | This study |
| AHO1096-ver2 | GACGGCACGGCCACGCGTTTAAAC | Modified from Nguyen *et al*. (2017) |
| AHO1097 | CCCGCCAGGCGCTGGGGTTTAAACACCG | Nguyen *et al*. (2017) |
| AHO1098-ver2 | CGAGACTTGCGTAAACTATTTTTAATTTG | Modified from Nguyen *et al*. (2017) |
| AHO1236 | TAAAGCTGCCACAAGAGGTATTTC | Nguyen *et al*. (2017) |
| AHO1237 | AGGTGATGCTGAAGCTATTGAAG | Nguyen *et al*. (2017) |


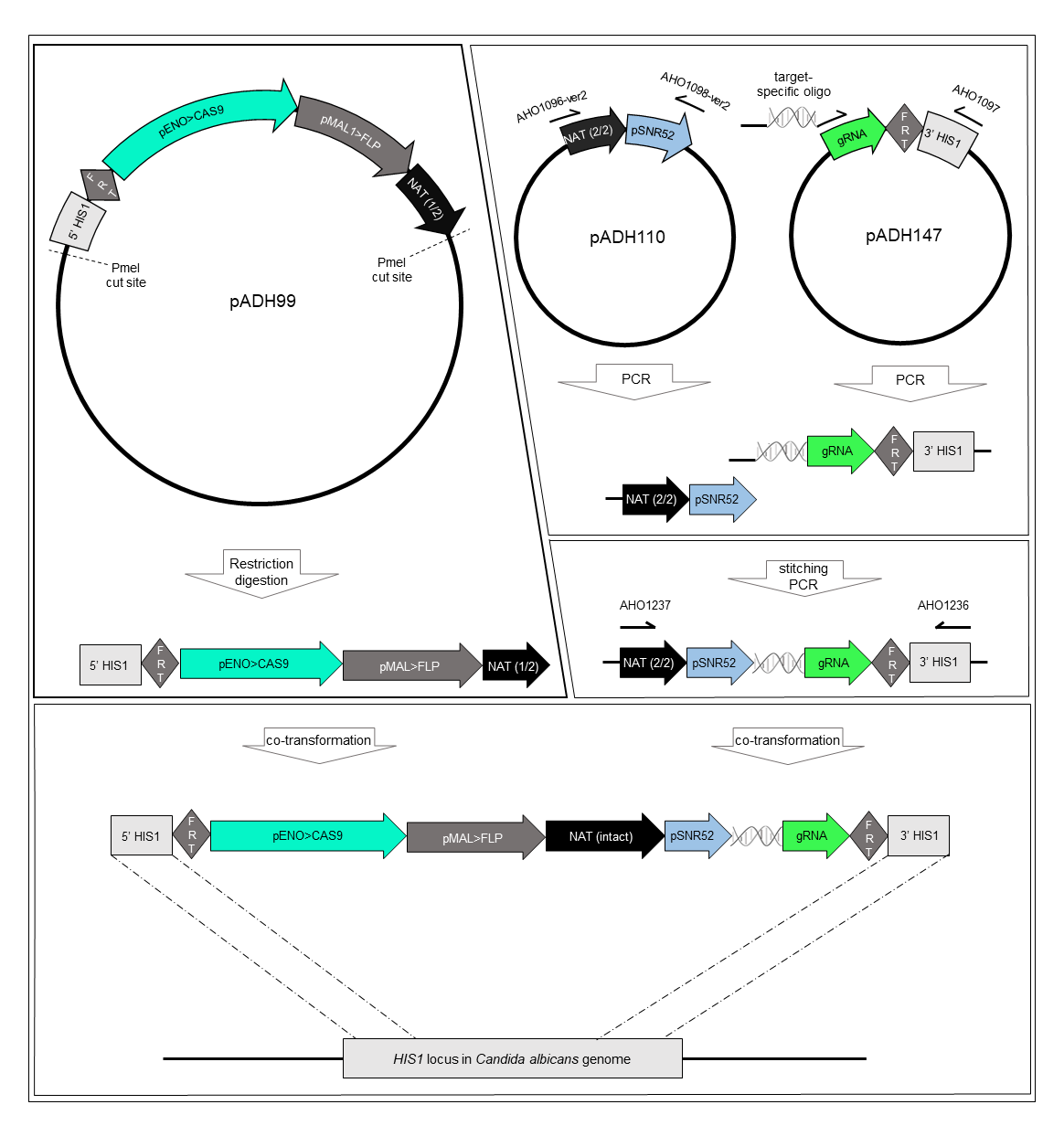


**A**

**B**

**C**

**D**

**Figure S1.** **Schematic representation of the preparation of the CRISPR *CAS9*-gRNA cassette for integration into the *Candida albicans* genome from Nguyen *et al*. (2017).** **A** – The *CAS9*-cassette is liberated via digestion of pADH99 with PmeI. **B** – The 5’ and 3’ end of the gRNA-cassette is prepared through PCR of pADH110 and pADH147. **C** – The full gRNA-cassette is created through stitching PCR of amplicons created in B. **D** – The *CAS9* and gRNA cassettes are co-transformed to create an intact *CAS9*-gRNA-cassette that integrates into the *HIS1* locus of the *Candida albicans* genome facilitated by homologous regions on each side of the cassette.

AHO1097 as reverse primer. The 3’ of the gRNA cassette (product size of 704 bp) was fused with the amplification product (1066 bp) of pADH110 with a stitching polymerase chain reaction with primers AHO1237 and AHO1236 (**Figure S1C**). The cassettes containing the *CAS9* as well as the gRNA is co-transformed into *C. albicans* to form an intact CAS9-gRNA cassette for integration into the *HIS1* locus (**Figure S1D**).

Donor DNA was designed to remove nearly the entire open reading frame of the target genes. A modification from the Nguyen and co-workers (2017) protocol entailed extending homologous regions to approximately 150 bp to 500 bp for deletion. In addition, homologous regions of approximately 500 bp were used for re-introduction of wild type genes (**Figure S2**).


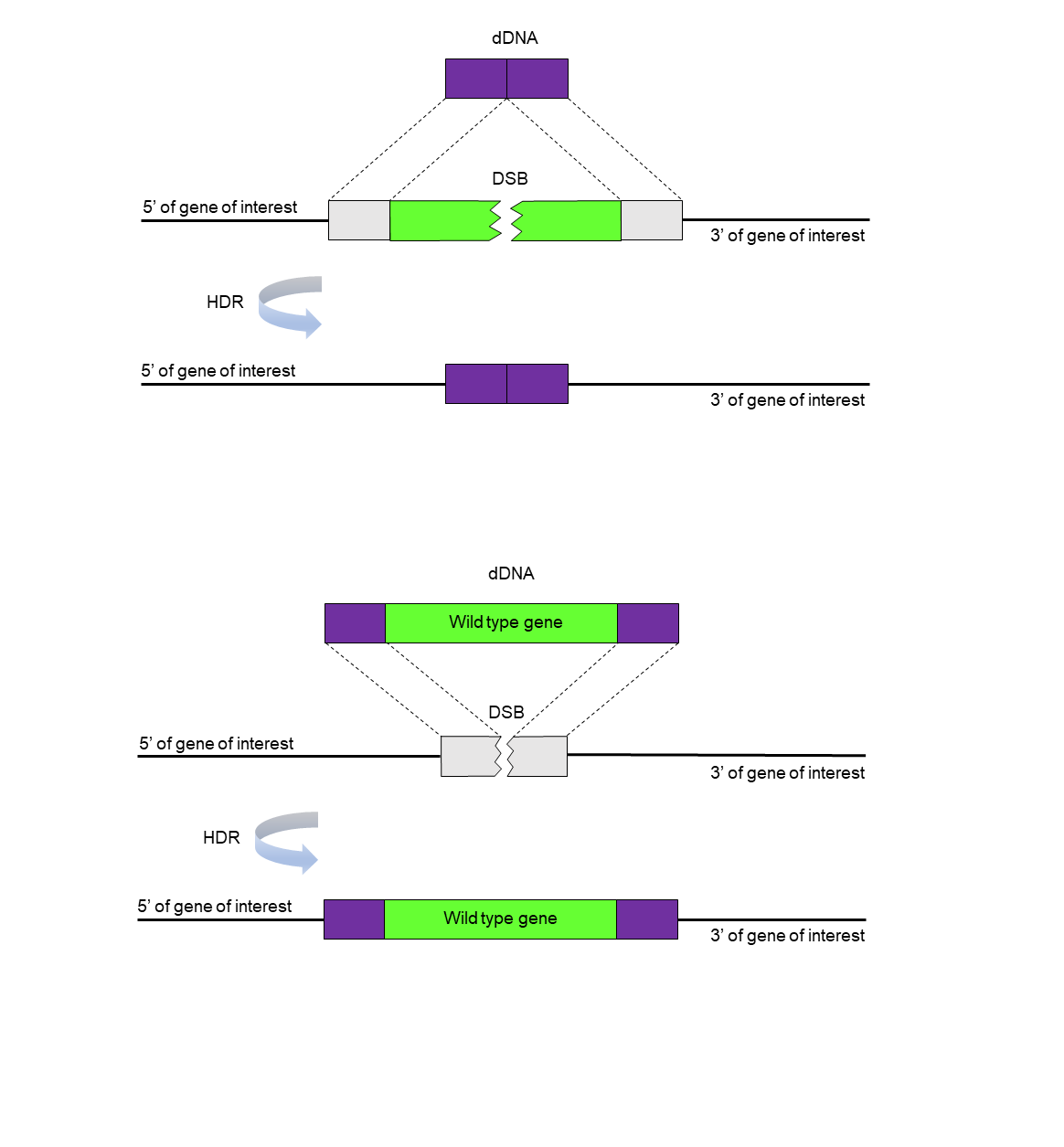


**Figure S2.** Schematic of homology directed repair (HDR) via addition of donor DNA (dDNA). **A** – donor DNA is designed to overlap with flanking regions of the gene of interest. After the formation of a double stranded break (DSB), dDNA is used to repair the DSB, facilitating the near entire removal of an open reading frame. **B** – a similar procedure is followed for the re-introduction of a gene, where the open reading frame is included in the dDNA.

**A**

**B**


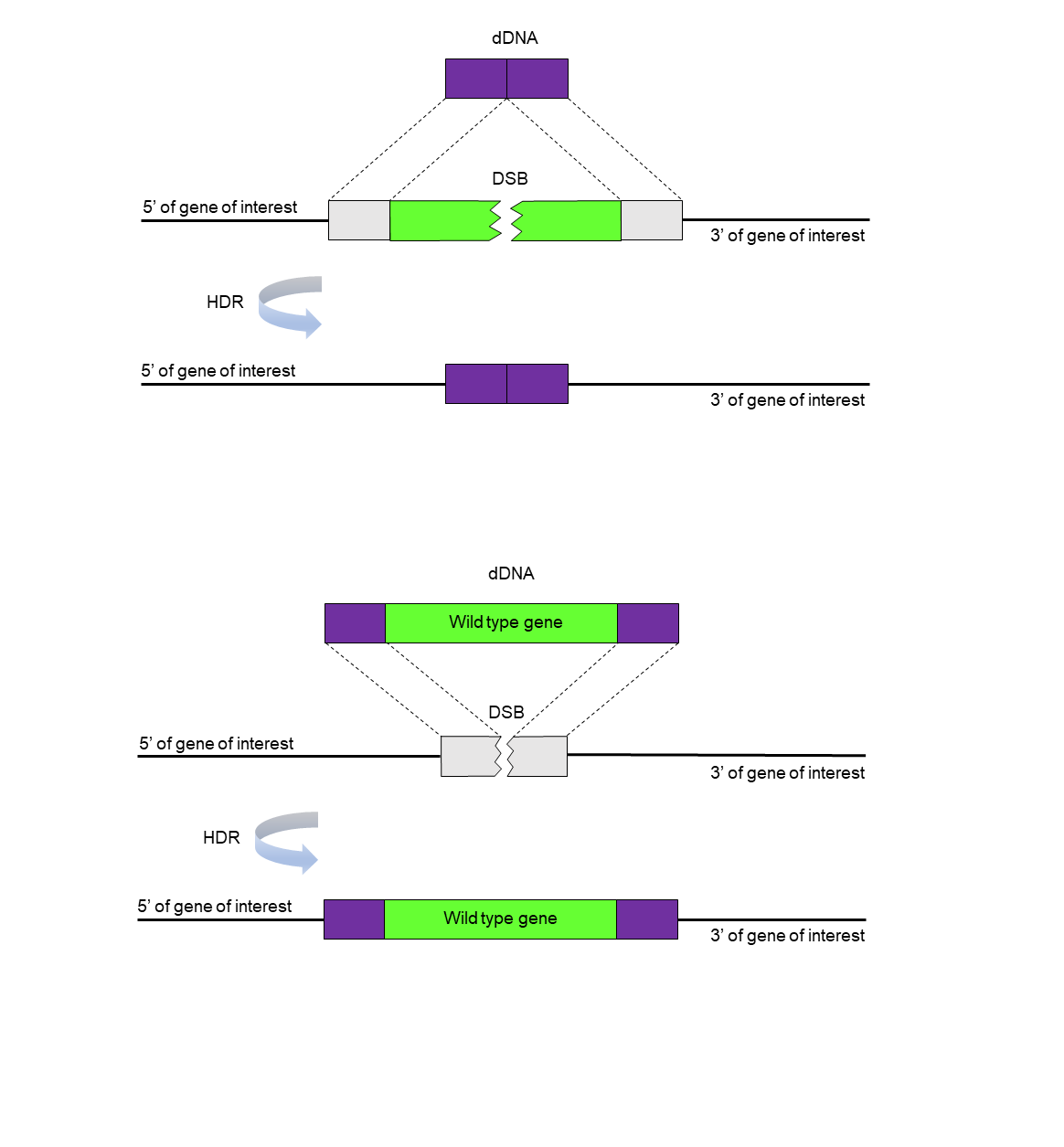


**Table S4** represents the information of the donor DNA constructed for the deletion of *SET3*. Genomic DNA was extracted with the *Quick*-DNA^TM^ Fungal/Bacterial Miniprep Kit (Zymo Research, USA) according to manufacturer’s specifications. Two fragments were amplified from the *C. albicans* SC5314 genome with fragment 1 located at the 5’ of the coding sequence fragment 2 at the 3’-end. Stitching PCR was performed to fuse fragment 1 and 2 using primer sets underlined in **Table S5** to produce complete donor DNA for deletion of the target genes.

**Table S4.** Unique CRISPR-site sequences without protospacer adjacent motif for deletion and add-back of *SET3*. Site-characteristics obtained in *Geneious*.

| Gene | Application | Sequence | On-site activity | Off-target activity |
| --- | --- | --- | --- | --- |
| *SET3* | Deletion | ATACTTATGGAGCTTTTCCG | 0.919 | 100.00% |
| *SET3* | Add-back | GCTTCTTTACAACTTTAGCA | 0.809 | 100.00% |

#### Transformation

*Candida albicans* SC5314 was inoculated in 5 mL YPD (10 g/L yeast extract, 20 g/L peptone, 20 g/L glucose) and incubated overnight with shaking (30°C). The overnight culture was inoculated into fresh YPD (ratio of 1:50) and allowed to reach an optical density (600 nm) of 0.5 to 0.8 after which cells were washed two times with sterile water (Nguyen *et al*., 2017). Cells were resuspended in 1/100 H_2_O of original volume. Un-purified digest of pADH99 and PCR products of gRNA cassette and dDNA were added to the washed cells together with 1 mL plate mix consisting of 875 µL 50% PEG 3350 (Sigma-Aldrich), 100 µL 10X TE buffer (100 mM Tris pH 7.4, 10 mM EDTA pH 8) and 25 µL 1M Lithium acetate (pH adjusted to 7 with acetic acid; Sigma-Aldrich) and incubated overnight at 30°C without shaking. Cells were heat-shocked at 44.6°C for 15 minutes, washed with sterile YPD and allowed to recover for 5 hours at 30°C with shaking. Recovered cells were plated onto YPD agar (15 g/L agar) plus 200 µg/mL nourseothricin (NTC, Jena Bioscience, Germany) and incubated for 2 to 3 days at 30°C to allow formation of colonies. Colony PCR was performed to amplify dDNA with primers SET3-2F and SET3-3R for *SET3*.

**Table S5.** Summary of donor DNA characteristics for deletion and add-back of *SET3*

| Gene | Application | Fragment | Forward primer | Reverse primer | Start position* | End position* | Coordinates of deletion/add-back | Size |
| --- | --- | --- | --- | --- | --- | --- | --- | --- |
| *SET3* | Deletion | 1 | SET3-2F | SET3-2R-overlap | -504 | -33 | -33 to 3039 | 3072 bp |
|  |  | 2 | SET3-3F-overlap | SET3-3R | 3039 | 3774 |  |  |
|  | Add-back | N/A | SET3-2F | SET3-3R | -504 | 3774 | -33 to 3039 | 4278 bp |

* Position from first bp of coding sequence.

Underlined primers were used for stitching polymerase chain reaction to fuse fragment 1 and fragment 2 of each gene for deletion.


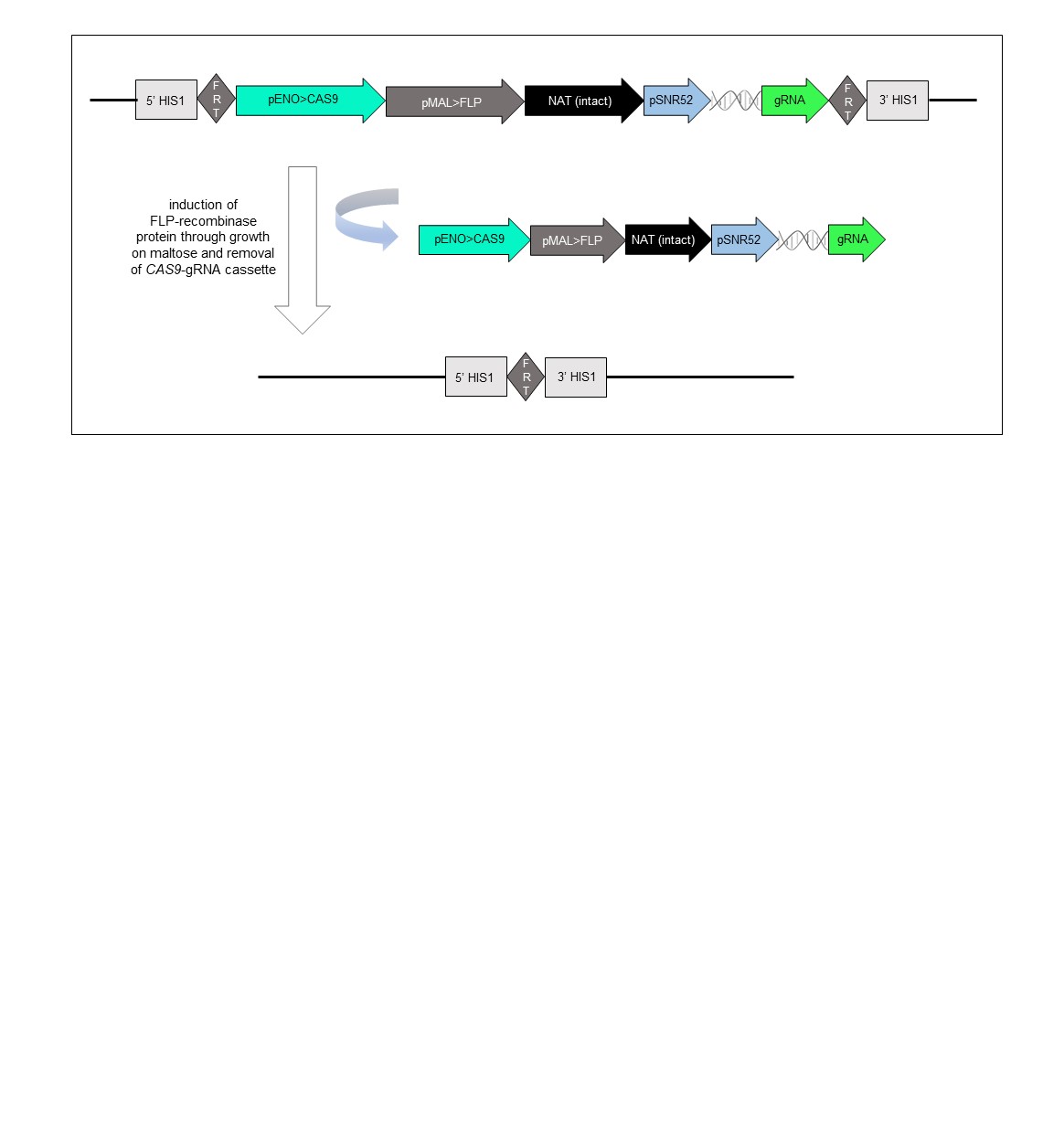


**Figure S3.** Schematic representation of the removal of the CRISPR *CAS9*-gRNA cassette from the *Candida albicans* genome.

- 1. *Removal of CAS9-gRNA cassette and knock-in of wild type gene*

After confirmation of deletion of the target gene, the CAS9-gRNA cassette was removed by inducing expression of a FLP/FRT recombinase system (under control of maltose-inducible promoter (**Figure 3**) through growth in Yeast Peptone Maltose media (10 g/L yeast extract, 20 g/L peptone and 20 g/L maltose) for 24h @ 30°C with shaking (200 rpm) (Nguyen *et al*., 2017). Cells were diluted in sterile H_2_0 and plated on YPD agar for 48h @ 30°C to allow formation of single colonies. After formation of colonies, growth on NTC was evaluated by streaking on YPD agar with 300 µg/mL NTC. The absence of growth in the presence of NTC indicates that the CAS9-gRNA cassette has been removed. After removal of the CAS9-gRNA cassette, a sequential round of transformation was performed as described above to re-introduce the wild type gene into its native locus (**Figure S2**).

1. **Results and Discussion**

For deletion with the CRISPR/Cas9 system, three components are required, a Cas9, a site-specific guide RNA (gRNA) and donor DNA (dDNA) for homology directed repair (HDR). The system used in this study makes use of a *CAS9* (from *Streptococcus pyogenes*, recognising a protospacer adjacent motif, PAM, of NGG) under the control of an *ENO1* promoter and gRNA (under the control of a *SNR52* promoter), carried on two separate cassettes that are co-transformed to integrate into one allele of the *HIS1* locus (**Figure 1**). Successful transformants are NTC resistant, accomplished by the *N*-acetyltransferase (NAT) marker (Nguyen *et al*., 2017). The *CAS9*-cassette contains the 5’-end of the NAT marker at its 3’-end, whereas the gRNA-cassette contains the 3’-portion on its 5’end. Successful integration of both cassettes yields a full NAT gene conferring resistance to nourseothricin. Expression of the Cas9p together with a unique guide RNA leads to a site-specific double stranded break. In addition, dDNA is co-transformed that is used for the repair of the double stranded break caused by the action of the Cas9. A graphical representation of this system can be seen in **Figure S4**.


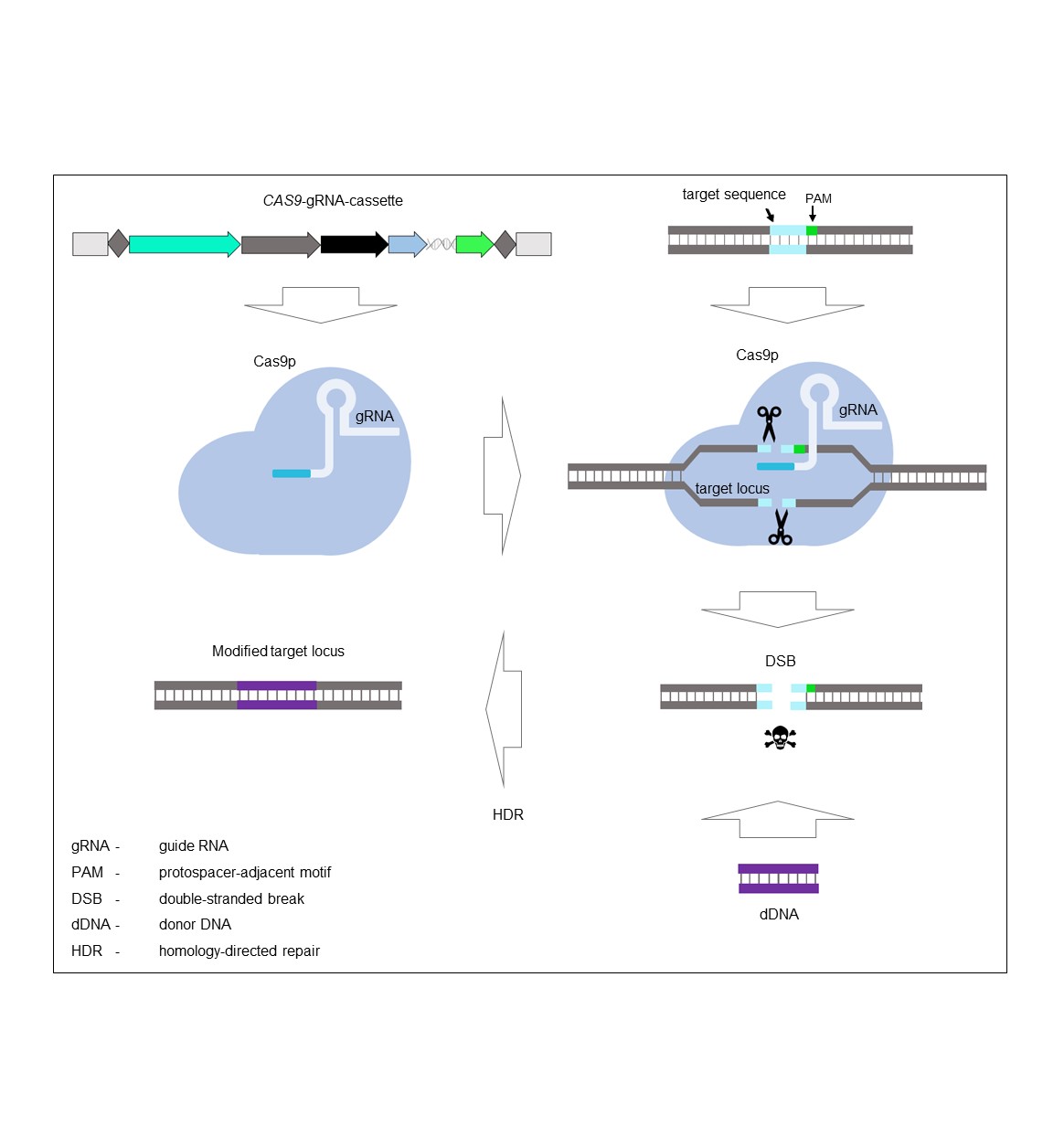


**Figure S4.** Schematic representation of the action of the CRISPR-Cas9 system published by Nguyen and co-workers (2017) after integration into the *Candida albicans* genome. Expression of the Cas9p and gRNA leads to the formation of a Cas9-gRNA complex. A unique gRNA sequence guides the complex to a target in the genome where the Cas9-gRNA complex forms a double stranded break (DSB). This DSB is repaired via homology directed repair with donor DNA (dDNA) that contains homologous regions to the target site of the DSB.

After confirmation of deletion with PCR, the *CAS9*-gRNA cassette was removed by induction of a maltose-inducible FLP/FRT recombinase system that renders the homozygous deletion mutants ready for another round of transformation to add the wild type gene back (Nguyen *et al*., 2017). By utilising this system, both copies of the wild-type gene can be reintroduced into the target locus. This second transformation event is also succeeded by the removal of the *CAS9*-gRNA cassette. The successful homozygous deletion and subsequent add-back of the wild type gene for *SET3* was achieved.

**References**

**Doench, J. G., Hartenian, E., Graham, D. B., Tothova, Z., Hedge, M., Smith, I., Sullender, M., Ebert, B. L., Xavier, R. J. *et al*. (2014).** Rational design of highly active sgRNAs for CRISPR-Cas9-mediated gene inactivation. *Nat Biotechnol* **32**, 1262-1267. doi: 10.1038/nbt.3026

**Hsu, P. D., Scott, D. A., Weinstein, J. A., Ran, F. A., Konermann, S., Agarwala, V., Li, Y., Fine, E. J., Wu, X., Shalem, O. *et al*. (2013).** DNA targeting specificity of RNA-guided Cas9 nucleases. *Nat Biotechnol* **31**, 827-832. doi: 10.1038/nbt.2647

**Muzzey, D., Schwartz, K., Weissman, J. S. and Sherlock, G. (2013).** Assembly of a phased diploid *Candida albicans* genome facilitates allele-specific measurements and provides a simple model for repeat and indel structure. *Genome Biology* **14**, R97. doi: 10.1186/gb-2013-14-9-r97

**Nguyen, N., Quail, M. M. F. and Hernday, A. D. (2017).** An efficient, rapid, and recyclable system for CRISPR-mediated genome editing in *Candida albicans*. *mSphere* **2**, e00149-17. doi: 10.1128/mSphereDirect.00149-17

**Skrzypek, M. S., Binkley, J., Binkley, G., Miyasato, S. R., Simison, M. and Sherlock, G.** “Candida Genome Database” http://candidagenome.org/ (accessed July 2018)
